# IL-1RN and IL-1*β* Polymorphism and ARV-Associated Hepatotoxicity

**DOI:** 10.1155/2018/4398150

**Published:** 2018-04-08

**Authors:** HariOm Singh, Dharmesh Samani, Vijay Nema, Manisha V. Ghate, R. R. Gangakhedkar

**Affiliations:** ^1^Department of Molecular Biology, National AIDS Research Institute, Pune, India; ^2^Department of Clinical Sciences, National AIDS Research Institute, Pune, India

## Abstract

The severity of hepatic injury depends upon cytokines. Previous studies associated *IL-1RN* allele 2 with *IL-1β* production. Hence, we examined the association of *IL-1 RN* and *IL-1β* polymorphisms with ARV-associated hepatotoxicity. Genotyping of *IL-1RN* (VNTR), *IL-1β* (-511C/T) polymorphisms was done in 162 HIV-infected patients, 34 with ARV hepatotoxicity, 128 without hepatotoxicity, and 152 healthy controls using PCR and PCR-RFLP method. The haplotypes 1T and 2C enhanced the risk for severe hepatotoxicity (OR = 1.41, *P* = 0.25; OR = 1.67, *P* = 0.31). *IL-1β-*511TT genotype significantly represented among tobacco using HIV-infected individuals compared to nonusers (OR = 3.74, *P* = 0.05). *IL-1β-*511TT genotype among alcohol users increased the risk for hepatotoxicity (OR = 1.80, *P* = 0.90). *IL-1β-*511CT and *-*511TT genotypes overrepresented in alcohol using HIV-infected individuals (OR = 2.29, *P* = 0.27; OR = 2.64, *P* = 0.19). *IL-RN* 2/2 and 1/3 genotypes represented higher in nevirapine using hepatotoxicity patients (OR = 1.42, *P* = 0.64, OR = 8.79, *P* = 0.09). *IL-1β-*511CT and *-*511 TT genotypes among nevirapine users enhanced the risk for severe hepatotoxicity (OR = 4.29, *P* = 0.20; OR = 1.95, *P* = 0.56). *IL-1β-*511CT and *-*511TT genotypes were overrepresented in combined nevirapine and alcohol using HIV-infected individuals as compared to nevirapine users and alcohol nonusers (OR = 2.56, *P* = 0.26; OR = 2.84, *P* = 0.24). *IL-1β-*511TT genotype with tobacco, alcohol, and nevirapine usage revealed a trend of risk for the development of ARV-associated hepatotoxicity and its severity.

## 1. Introduction

Drug-induced adverse effects have been reported with the use of all antiretroviral (ARV) drugs [1]. Hepatotoxicity is a relatively common adverse drug reaction (ADR) leading to the treatment interruptions in human immunodeficiency virus- (HIV-) infected patients on different drug regimens [2], with consequent immunologic compromise [3]. The severity of the hepatic injury and drug-induced liver injury (DILI) depends upon the toxic molecules (reactive oxygen species) and the counterbalance of cytokines (TNF-*α*, IL-1*β*, IL-10, and IL-1RA) [4–6]. 10.8% in the efavirenz-treated group while 8.9% in the nevirapine-treated group reported having grade 3 or 4 hepatotoxicity [7]. While in another report, nevirapine use was associated with a higher incidence of hepatotoxicity than efavirenz use [8]. Nagpal et al. [9] reported a prevalence of nevirapine-induced hepatotoxicity (3.19%) in HIV-infected individuals in India. The activity of drug metabolizing enzymes was found to be altered in some infectious and inflammatory states such as bacterial pneumonia, viral infections, surgery, and trauma [10]. The effect of infection on the drug metabolizing enzymes is carried by the few cytokines such as *IL-1β*, *IL6*, tumor necrosis factor alpha (*TNF-α*), and interferon (*INF*) *α* or *γ* [11, 12]. IL-1 receptor antagonist (IL-1RA) restricted the induction of HIV replication in vitro [13–15], and the treatment with ARV drugs resulted in an increased level of circulating IL-1RA [16].

Interleukins (ILs) are proinflammatory cytokines produced by leukocytes, macrophages, monocytes, and epithelial cells. The IL-1 family consists of eleven members including IL-1 alpha (IL-1*α*), IL-1 beta (IL-1*β*), and IL-1 receptor antagonist (IL-1RA) [17]. IL-1*α* and IL-1*β* are the most influencing proinflammatory cytokines, and IL-1RN is a naturally occurring anti-inflammatory cytokine [18, 19]. The IL-1RA also regulates biological activity of IL-1*α* and IL-1*β* [19–21]. ILs promote the interaction of endothelial cells with circulating leukocytes and induce the proliferation of monocytes and macrophages.

IL-1 cytokine cluster spanning ~430 kb region which is located on chromosome 2q13-21, consists of *IL-1α*, *IL-1β*, and *IL-1RN* genes [22]. IL-1*β* plays a pivotal role in driving inflammation by binding to the IL-1 receptor to induce more proinflammatory cytokines. The proinflammatory cytokine IL-1*β* and its antagonist IL-1RA are strongly induced by infection and are encoded by polymorphic genes [23]. IL-1*β* production is tightly regulated by two steps: induction of pro*-IL-1β* gene expression and caspase-1-mediated pro-IL-1*β* cleavage [24]. Polymorphisms have been reported in all the three genes [25]. *IL-1RN* gene contains 86-bp variable number tandem repeat (VNTR) polymorphism in intron 2 region [26]. *IL-1RN* alleles 1 and 2 are the most common, while other alleles occur at a combined frequency of <5%. The role of *IL-1RN* (VNTR) has been studied in the development of inflammatory disorders [27–29]. Witkin et al. [30] suggested that the presence of *IL-1RN* 2/2 homozygous genotype reduced the viral load (*P* < 0.05) in the treatment-naive HIV-infected individuals. There was no relation between *IL-1RN* alleles and CD4 levels. Plasma IL-1RA concentrations were higher with borderline significance (*P* = 0.058) in treatment-naive HIV-infected adults [31].

In *IL-1β*, three biallelic polymorphisms are reported representing C-T base transitions, at positions −511, −31, and +3954 bp from the transcriptional site. *IL-1β*-511C/T polymorphism has been associated with various inflammatory diseases and in linkage disequilibrium with the *IL-1RN* polymorphism [32].

*IL-1β* promoter (-511C/T) and exon 5 (+3953C/T) polymorphisms may influence the gene expression and also found to be associated with various inflammatory diseases [33, 34]. Asensi et al. [35] suggested that *IL-1β* 3954T allele was less frequent in the patients with lipodystrophy syndrome compared with those without (17.8% versus 27.0%, *P* = 0.03). However, the *IL-1β* 3954T allele was associated in patients with lipodystrophy syndrome who are under highly active antiretroviral therapy (HAART) (*P* = 0.02). The carriage alleles of *IL-1α-*889, *IL-1β*-3953, and *IL-1β*-3′UTR did not differ between HIV-associated dementia (HAD) patients and controls [36].

Till now, the association of *IL-1RN* (VNTR) and *IL-1β* (-511C/T) polymorphisms has not been described with the severe hepatotoxicity and its prevalence in HIV-infected individuals on NNRTI-containing ART and healthy controls. Hence, the present study is intended to evaluate the association of *IL-1RN* (VNTR) and *IL-1β* (-511C/T) polymorphism with severe hepatotoxicity and its prevalence in HIV-infected individuals and healthy controls. Its interaction with environmental factors was also analyzed.

## 2. Material and Methods

### 2.1. Participants

This is a case-control study, carried out from November 2012 to February 2015. A total of 162 HIV-infected individuals were recruited. Out of that, 34 HIV-infected individuals with hepatotoxicity (grade III/IV) under NNRTI-containing ART regimen, 128 without hepatotoxicity-confirmed liver function test (LFT), and age-matched 152 healthy individuals were consecutively recruited from outpatient clinics of National AIDS Research Institute, Pune. Nevirapine was given in the standard dosage of 200 mg twice a day. Nevirapine was given in the standard dosage of 200 mg twice a day. In HIV-infected individuals with hepatotoxicity cases, having hepatitis B, hepatitis C, tuberculosis, and concurrent untreated opportunistic infections, immune reconstitution syndrome and under any other known hepatotoxic drugs were excluded from the case group. In the group of HIV-infected individuals without hepatotoxicity, individuals having evidence of hepatotoxicity, hepatitis B, hepatitis C, tuberculosis, and receiving any other known hepatotoxic drugs were excluded. Similarly, 152 (those from the same family were excluded), free of Hepatitis B and C and tuberculosis, age-matched and serum negative individuals from HIV-ELISA test were recruited. Clinical data were obtained by questionnaire, personal interviews, and review of case records. LFT was done to evaluate the status of the liver enzyme. Total bilirubin > 3.22 mg/ml, SGOT > 93.8 U/ml, SGPT > 229.5 U/ml, and alkaline phosphatase > 550.8 U/ml for male hepatotoxicity cases and total bilirubin > 3.22 mg/ml, SGOT > 163.2 U/ml, SGPT > 173.4 U/ml, and alkaline phosphatase > 550.8 U/ml for female hepatotoxicity were considered as cases. Total bilirubin < 1.24 mg/ml, SGOT < 32 U/ml, SGPT < 34 U/ml, and alkaline phosphatase < 108 U/ml for male and female were considered as HIV-infected control. Estimation of the CD4 count was done by fluorescence-activated cell sorting (FACS). ELISA for hepatitis C and HBsAg testing was performed using Ortho HCV ELISA test system (Ortho Clinical Diagnostics, Buckinghamshire, UK) and Murex HBsAg Confirmatory Version 3 (DiaSorin, Dartford, UK) ELISA. The status of tobacco and alcohol usage was also recorded in the questionnaire. The study was approved by the institutional ethics committee board and informed written consent was taken from all eligible participants.

### 2.2. DNA Extraction

A 2 ml peripheral blood sample was collected and stored at −70°C before DNA extraction. Extraction of genomic DNA was done using AxyPrep Blood Genomic DNA Miniprep Kit (Axygen Biosciences, Union City, CA, USA) according to the protocol given by the manufacturer.

### 2.3. Genotyping

#### 2.3.1. *IL-1RN* (86 bp VNTR) Polymorphism

*IL-1RN* (86 bp repeats) polymorphism was determined by polymerase chain reaction (PCR) analysis as described by Tarlow et al. [26]. The reaction mixture for PCR was prepared in a total volume of 25 *μ*l with 10 pmol primers, 10x standard Taq buffer, genomic DNA (100–150 ng), 2.5 mM deoxynucleotide triphosphates (dNTPs), and one unit of Taq DNA polymerase (New England Biolabs, USA). The reaction conditions for *IL-1RN* (VNTR) were initial denaturation at 95°C for 5 min, followed by 35 cycles of denaturation at 95°C for 30 sec, annealing at 58°C for 30 sec, extension at 72°C for 30 sec, and a final extension at 72°C for 10 min. The amplified PCR product of *IL-1RN* was genotyped on 3% agarose gel. The genotypes were determined as allele 1, 410 bp (four repeats); allele 2, 240 bp (two repeats); allele 3, 500 bp (five repeats); allele 4, 325 bp (three repeats); and allele 5, 595 bp (six repeats) [37].

#### 2.3.2. *IL-1β* (-511C/T) Polymorphism

*IL-1β* (-511C/T) polymorphism was assessed by PCR followed by the restriction digestion (PCR-RFLP) method. PCR reaction was prepared in a total volume of 25 *μ*l with primers (10 pmol) as described by Rad et al. [38], 10x standard Taq buffer, genomic DNA (100–150 ng), 2.5 mM deoxynucleotide triphosphates, and 1 unit of Taq DNA polymerase (New England Biolabs, USA). PCR conditions were as follows: initial denaturation at 94°C for 3 min, followed by 35 cycles of denaturation at 95°C for 30 sec, annealing at 51°C for 30 sec, extension at 72°C for 45 sec, and a final extension at 72°C for 7 min. Further, the amplified product was digested using *AvaI* restriction enzyme (MBI Fermentas Inc., Hanover, MD, USA). The genotype of *IL-1β* (-511C/T) was determined on 10% polyacrylamide gel after staining with ethidium bromide with reference to molecular weight markers. Genotypes were assessed as follows: 304 bp for TT, 190 and 114 bp for CC and 304, and 190 and 114 bp for CT. All PCR reactions were performed in a Veriti 96-well thermal cycler (Applied Biosystems, CA, USA). To avoid the data discrepancy in genotyping, 20% of samples from patients and controls were regenotyped by another laboratory personnel, and also 10% of samples were assessed by sequencing. No inconsistency was noticed with the previously recorded genotypes.

### 2.4. Data Analysis

The mean age variable was considered with an appropriate standard deviation. Deviations from Hardy-Weinberg equilibrium in control were assessed by chi-square (*χ*^2^) goodness-of-fit test. Chi-squared test was also used to compare genotype frequencies in HIV-infected individuals with hepatotoxicity, without hepatotoxicity, and healthy controls. SHEsisPlus online analysis tool was used to compare haplotype frequency between HIV-infected individuals with hepatotoxicity, without hepatotoxicity, and healthy controls [39–41]. Odds ratios (ORs) and 95% confidence interval (CI) were determined by multivariate unconditional binary logistic regression using SPSS software version 17.0 (SPSS Inc. Released 2008. SPSS Statistics for Windows, Version 17.0. Chicago: SPSS Inc.) Data was analyzed for the two-sided statistical significance, and a *P* value less than 0.05 was considered for the statistically significant difference. Linkage disequilibrium (LD) was assessed between both the loci by calculating the relative LD value (D') as D′ = Dij/Dmax [42]. The Dij values were compared between HIV-infected individuals with hepatotoxicity versus without hepatotoxicity, HIV-infected individuals with hepatotoxicity versus healthy controls, and HIV-infected individuals without hepatotoxicity versus healthy controls by comparison of confidence intervals.

## 3. Results

Thirty-four HIV-infected individuals with hepatotoxicity, 128 without hepatotoxicity, and 152 healthy controls were included in the study. The mean age difference between all study groups were 35.14 ± 8.96, 39.29 ± 1.34, and 36.75 ± 8.50 years, respectively. Characteristics of HIV-infected individuals with, without hepatotoxicity, and healthy controls are shown in Table 1.

### 3.1. *IL-1RN* (VNTR) and *IL-1β* (-511C/T) Polymorphism and HIV-Infected Individuals with Hepatotoxicity

In single and both locus model, none of the genotypes/alleles of *IL-1RN* and *IL-1β* polymorphism was associated with severe hepatotoxicity. *IL-1RN* 1/3 genotype and allele 3 showed higher risk for severe hepatotoxicity (5.9% versus 1.6%, OR = 3.16, 95% CI: 0.29–34.43, *P* = 0.56 and 2.94% versus 1.17%, OR = 2.35, 95% CI: 0.27–17.89, *P* = 0.68, resp.). The frequency of *IL-1RN* 1/2 genotype and allele 2 was lower in HIV-infected individuals with hepatotoxicity compared to without hepatotoxicity (29.4% versus 39.9%, OR = 0.62, 95% CI: 0.24–1.56 and 23.52% versus 28.51%, OR = 0.77, 95% CI: 0.39–1.50, resp.). The frequency of *IL-1RN* 2/2 genotype was observed to be almost similar in both the groups (8.8% versus 7.8%).

In the *IL-1β* (-511C/T) polymorphism, the frequency of -511CT, -511TT genotypes, and -511 T allele was found to be almost similar in both the groups (41.2% versus 47.7%, 38.2% versus 35.9%, and 58.82% versus 59.76%, resp.).

The frequency of *IL-1RN* and *IL-1β* genotypes/alleles did not differ between HIV-infected individuals with hepatotoxicity and healthy controls. However, *IL-1RN* 1/3 genotype and allele 3 were overrepresented in HIV-infected individuals with hepatotoxicity compared with healthy controls (5.9% versus 2.0%, OR = 2.39, 95% CI: 0.37–15.33, *P* = 0.69 and 2.94% versus 1.64%, OR = 1.62, 95% CI: 0.20–8.57, *P* = 0.93, resp.) (Table 2).

### 3.2. *IL-1RN* (VNTR) and *IL-1β* (-511C/T) Polymorphism and HIV-Infected Individuals

The genotype/allele frequencies of *IL-1RN* (VNTR) and *IL-1β* (-511C/T) polymorphisms in HIV-infected individuals and healthy controls are shown in Table 3. The genotype distributions of *IL-1RN* (VNTR) and *IL-1β* (-511C/T) polymorphism followed Hardy-Weinberg equilibrium in the healthy control population (*P* = 0.67 and 0.12). In single and both locus model, the frequency of *IL-1RN* and *IL-1β* genotypes/alleles did not differ significantly between HIV-infected individuals and healthy controls. However, the prevalence of *IL-1RN* 1/4 genotype and allele 4 was observed to be higher in HIV-infected individuals compared with healthy controls (2.3% versus 0.7%, OR = 3.40, 95% CI: 0.30–87.20 and 1.56% versus 0.32%, OR = 4.59, 95% CI: 0.48–108.86, resp.). The frequency of *IL-1RN* 1/2, 2/2, and 1/3 genotypes and alleles 2 and 3 presented almost similarly in HIV-infected individuals and healthy controls (39.9% versus 40.1%, 7.8% versus 10.5%, and 1.6% versus 2.0% and 28.51% versus 31.25% and 1.17% versus 1.64%, resp.). The frequency of *IL-1β*-511CT genotype marginally differed in HIV-infected individuals compared with healthy controls (47.7% versus 40.8%).

### 3.3. *IL-1RN* (VNTR) and *IL-1β* (-511C/T) Polymorphisms and HIV Disease Stages

Genotype frequencies of *IL-1RN* and *IL-1β* polymorphisms in patients with different HIV disease stages and healthy controls are presented in Table 4. *IL-1RN* 2/2 genotype was less prevalent in patients with advanced HIV disease stage compared to healthy controls (5.6% versus 11.0%, OR = 0.42, 95% CI: 0.11–1.52, *P* = 0.19), while the frequency of *IL-1β*-511CT genotype was observed higher in patients with early HIV disease stage compared to healthy controls (57.9% versus 40.8%, OR = 2.66, 95% CI: 0.50–14.21, *P* = 0.25; 47.4% versus 40.8%, OR = 1.46, 95% CI: 0.56–3.81, *P* = 0.44).

### 3.4. Gene-Gene Interaction

In the gene-gene interaction analysis, the haplotype frequencies of *IL-1RN* (VNTR) and *IL-1β* (-511C/T) polymorphisms in HIV-infected individuals with hepatotoxicity, without hepatotoxicity, and healthy controls are shown in Table 5. The linkage disequilibrium (LD) values and comparison of LD values (Dij) between HIV-infected individuals with hepatotoxicity versus without hepatotoxicity, HIV-infected individuals with hepatotoxicity versus healthy controls, and HIV-infected individuals versus healthy controls have no significant difference in both the genes (0.32, 0.32, and 0.39). It was expected that there might be the additive or synergistic effect of these variations with the development of ARV-associated hepatotoxicity and its severity.

Haplotype 1C (*IL-1RN *^∗^1/*IL-1β *^∗^C) was taken as reference. Haplotype 2T (*IL-1RN *^∗^2/*IL-1β *^∗^T) was underrepresented in cases of severe hepatotoxicity compared with HIV-infected individuals (15% versus 22%, OR = 0.60, 95% CI: 0.29–1.25, *P* = 0.17). The haplotypes 1T and 2C (*IL-1RN *^∗^1/*IL-1β *^∗^T and *IL-1RN *^∗^2/*IL-1β *^∗^C) showed risk for the cases of severe hepatotoxicity (43% versus 35%, OR = 1.41, 95% CI: 0.79–2.36, *P* = 0.25 and 9% versus 5%, OR = 1.67, 95%CI: 0.61–4.53, *P* = 0.31, resp.).

The frequency of haplotype 2T (*IL-1RN *^∗^2/*IL-1β *^∗^T) differed between HIV-infected individuals with hepatotoxicity and healthy controls with borderline significance, and the risk was reduced for the cases of severe hepatotoxicity (15% versus 25%, OR = 0.51, 95% CI: 0.25–1.02, *P* = 0.07).

The haplotypes frequency of *IL-1RN*/*IL-1β* polymorphism did not differ between HIV-infected individuals and healthy controls.

### 3.5. *IL-1RN* (VNTR) and *IL-1β* (-511C/T) Polymorphism and Environmental Factors

In the gene-environment interaction analysis, the effect of tobacco and alcohol usage in the HIV-infected individuals was analyzed with respect to *IL-1RN* (VNTR) and *IL-1β* (-511C/T) polymorphism. The frequency of *IL-1β* carriage *-*511TT genotype did not differ between tobacco using cases of severe hepatotoxicity and nonusers (42.9% versus 37.0%, OR = 0.75, 95% CI: 0.06–9.47, *P* = 0.78). However, the frequency of *IL-1β* carriage *-*511TT genotype was significantly higher in tobacco using HIV-infected individuals compared with nonusers (52.4% versus 29.1%, OR = 3.74, 95% CI: 0.97–15.57, *P* = 0.05).

*IL-1β*-511CT and -511TT genotypes among alcohol users increased the risk of severe hepatotoxicity (42.9% versus 40.7%, OR = 1.64, 95% CI: 0.10–51.13, *P* = 0.84 and 42.9% versus 37.0%, OR = 1.80, 95% CI: 0.11–56.75, *P* = 0.90, resp.). Similarly, *IL-1β*-511CT and -511 TT genotypes and alcohol usage showed increased risk to develop ARV-associated hepatotoxicity (48.8% versus 45.9%, OR = 2.29, 95% CI: 0.61–9.28, *P* = 0.27 and 41.9% versus 34.1%, OR = 2.64, 95% CI: 0.68–11.06, *P* = 0.19, resp.) (Table 6).

*IL-1RN* (VNTR) and *IL-1β* (-511C/T) polymorphisms were studied to determine the influence of NNRTI regimen and a combination of the regimen with alcohol usage in the risk to develop ARV-associated hepatotoxicity and its severity.

*IL-1RN* 2/2 and 1/3 showed an elevated risk in cases of severe of hepatotoxicity compared to without hepatotoxicity among nevirapine users (13.0% versus 8.6%, OR = 1.42, 95% CI: 0.33–6.20, *P* = 0.64 and 8.7% versus 0.86%, OR = 8.79, 95% CI: 0.71–109.16, *P* = 0.09, resp.). Additionally, *-*511CT and *-*511TT genotypes of *IL-1β* indicated higher risk for the cases of hepatotoxicity with efavirenz usage compared with nonusers (45.5% versus 16.7%, OR = 4.29, 95% CI: 0.47–39.40, *P* = 0.20 and 27.3% versus 58.3%, OR = 1.95, 95% CI: 0.20–19.29, *P* = 0.56, resp.) (Table 7).

*IL-1RN 1/2* and *IL-1β-*511TT genotypes enhanced the risk for the cases of severe hepatotoxicity among nevirapine usage (30.4% versus 27.3%, OR = 1.70, 95% CI: 0.26–11.84, *P* = 0.81 and 43.5% versus 27.3%, OR = 2.50, 95% CI: 0.24–28.86, *P* = 0.68, resp.). Similarly, the frequency of *IL-RN* 1/2 and *IL-1β-*511CT genotypes were found to be higher among nevirapine using HIV-infected individuals compared with efavirenz users (41.4% versus 25.0% OR = 2.11, 95% CI: 0.45–11.02, *P* = 0.46 and 50.0% versus 16.7%, OR = 4.83, 95% CI: 0.59–45.70, *P* = 0.20, resp.) with increased risk for the development of ARV-associated hepatotoxicity (Table 8).

The frequency of *IL-1β* carriage*-*511TT genotype was found to be higher in both nevirapine and alcohol using cases of hepatotoxicity compared to nevirapine users and alcohol nonuser cases (60% versus 38.88%, OR = 1.29, 95% CI: 0.05–47.87, *P* = 0.63). Similarly, *IL-1β-*511CT and *-*511TT genotypes were overrepresented among both nevirapine and alcohol using HIV-infected individuals as compared with nevirapine users and alcohol nonusers (54.05% versus 49.37%, OR = 2.56, 95% CI: 0.59–12.68 and 37.84% versus 31.65%, OR = 2.84, 95% CI: 0.60–14.71, *P* = 0.24, resp.). The frequency of *IL-RN*1/2 and *IL-1β-*511TT genotypes were higher in combined efavirenz and alcohol using HIV-infected individuals compared with efavirenz users and alcohol nonusers (33.33% versus 20.00, OR = 2.67, 95% CI: 0.09–129.92, *P* = 1.00 and 66.67% versus 50.00%, OR = 4.00, 95% CI: 0.16–174.72, *P* = 0.68, resp.) (Table 9).

## 4. Discussion

This is the first study that simultaneously examines the impact of genetic polymorphisms in the two loci of IL-1 cluster to develop ARV-associated hepatotoxicity and its severity. The IL-1RA is an immunologic regulator that competes with other IL-1 family members (IL-1*α* and IL-1*β*) for the IL-1 receptor in the target cells and acting as its negative regulator with an anti-inflammatory effect [18, 43, 44]. In response to inflammation, proinflammatory cytokines (IL-1*β* and TNF-*α*) are produced to control infections [45, 46]. The proinflammatory response is downregulated by cytokine transforming growth factor-*β* (TGF-*β*, IL-10) and specifically in the case of IL-1, IL-1RA to terminate the immune response and limit the potential for immunopathology [18]. An interindividual variation in the IL-1 family of genes seen not only in the susceptibility to HIV infection but also in the progression of HIV-1 [31, 35, 36]. These genetic variations may also influence the gene expression.

In the present study, the frequency of *IL-1RN* (VNTR) and *IL-1β* (-511C/T) genotypes in healthy controls was comparable with a study carried out by Kesarwani and Mittal from North India and differed from other reports available from North India [37, 47–49]. In the present study, *IL-1RN* (VNTR) and *IL-1β*-511C/T polymorphisms were neither associated with severe hepatotoxicity and nor its genotypes distribution differed significantly between HIV-infected individuals and healthy controls. *IL-1RN* allele 2 has been related to increased production of IL-1RA and IL-1*β* and decreased the production of IL-1*α* [43]. There are no differences in the IL-1*β* pattern in HIV-1-infected compared to noninfected monocytes [50–52]. The distribution of *IL-1RA* genotypes found to be similar in the HIV-seropositive women and HIV-seronegative women [30, 53]. A low prevalence of *IL-1RN* allele 2 in a Black African people and an African-American population has also been previously reported [54, 55].

Multivariate loci contribute to the disease susceptibility rather than the polymorphism on single locus which indicates that haplotype study is more important to estimate the effect of polymorphisms on the gene expression [56]. Therefore, haplotype analysis accurately provides information about the interaction among the adjacent genes. In our study, haplotypes 1T and 2C showed a higher risk of severe hepatotoxicity (OR = 1.41, *P* = 0.25 and OR = 1.67, *P* = 0.31, resp.). It was known that haplotype risk was equal to the summation of individual risks of its constituent alleles.

In the current study, we also analyzed the distribution of IL*-1RN* and *IL-1B* polymorphisms in HIV disease stages to modulate the risk of HIV disease advancement. The presence of *IL-1β*-511CT genotype increased the risk of disease advancement in patients with early and advanced HIV disease stages compared to healthy controls (OR = 2.66, *P* = 0.25; OR = 1.46, *P* = 0.44).

Studies suggested that the gene-environment interaction determines the etiology of the disease [57, 58]. However, the selection criteria for case-control association studies with the environmental influences and controls must be matched with cases in the population. Otherwise, it can lead to false interactions [59]. In such cases, the case-only method may be used to study gene-environment interaction under the assumption of the independence between exposure and genotype in the population [60, 61]. Therefore, we adopted the case-only study design to describe the influence of the risk in the development of ARV-associated hepatotoxicity and its severity with tobacco, alcohol, and drug usage. A study reported that heavy alcohol consumption has a negative impact on the CD4 cell count in the HIV-infected people who are not having combinational antiretroviral therapy [62]. A study conducted in HIV-infected population investigated that a decreased response to ART in smoking HIV-infected women. In the present study, *IL-1β* carriage*-*511TT genotype among alcohol users increased the risk in cases of severe hepatotoxicity (OR = 1.80, *P* = 0.90). Similarly, *IL-1β-*511TT genotype among alcohol users increased the risk for the development ARV-associated hepatotoxicity in HIV-infected individuals (OR = 2.64, *P* = 0.19). It supports the idea that individuals with *IL-1β*-511TT genotype are more prone to alcohol-related hepatotoxicity.

The *IL-1β* carriage *-*511TT genotype among nevirapine users was likely to be associated with cases of severe hepatotoxicity (43.5% versus 27.3%, OR = 2.50, *P* = 0.68). Also, *IL-1β-*511CT genotype among nevirapine using HIV-infected individuals enhanced the risk for the development of ARV-associated hepatotoxicity (OR = 4.83, *P* = 0.20). *IL-1β-*511TT genotype among combined nevirapine and alcohol users increased the risk for the development of ARV-associated hepatotoxicity in HIV-infected individuals (OR = 2.84, *P* = 0.24). It suggested that individuals with *IL-1β*-511TT genotype are likely to be at risk of alcohol- and drug-induced hepatotoxicity.

## 5. Conclusion

*IL-1β*-511TT genotype may influence the development of ARV-associated hepatotoxicity and its severity in alcohol and nevirapine users independently and in combination. The present study suggested that *IL-RN* (VNTR) and *IL-1β-*511C/T polymorphisms should be explored simultaneously further in cases of severe hepatotoxicity in larger sample size with other population. Also, further studies of other inflammatory response related gene (*TNF-α*, *IL-2*, *IL-10*, and *TGF-β*) are required to understand the termination of immune response and limit the potential for immunopathology. *IL-1β* polymorphism may reflect the host immune status as well as the control over natural disease and may also affect the virological response to antiviral treatment. Genotyping of *IL-1RN* (VNTR) and *IL-1β* 511C/T polymorphism may be beneficial to predict the immune defense against infection and a prolonged Th1 cell-mediated immune response.

## Figures and Tables

**Table 1:** Characteristics of HIV-infected individuals with hepatotoxicity, without hepatotoxicity, and healthy controls.

Subjects	HIV-infected individuals with hepatotoxicity (grade III and IV)	HIV-infected individuals without hepatotoxicity	Healthy controls
Number	*N* = 34	*N* = 128	*N* = 152
Mean age (range)	35.14 ± 8.96	39.29 ± 1.34	36.75 ± 8.50
Females	16	44	40
Males	18	84	112
*NNRTI regimen*			
Efavirenz (*N* = 23)	11 (47.8)	12 (52.2)	NA
Nevirapine (*N* = 139)	23 (16.50)	116 (83.50)	NA
*Alcohol habit* (*N* = 162)			
User (*N* = 50)	7 (14.0)	43 (86.0)	0
Nonuser (*N* = 112)	27 (24.11)	85 (75.89)	0
*Tobacco habit* (*N* = 162)			
User (*N* = 49)	7 (14.28)	42 (85.71)	0
Nonuser (*N* = 113)	27 (23.89)	86 (76.11)	0
*CD4+ status* (*N* = 162)			
<200 (*N* = 92)	16 (17.39)	76 (82.61)	NA
201–350 (*N* = 50)	17 (34.0)	33 (66.0)	NA
>350 (*N* = 20)	1 (5.0)	19 (95.00)	NA

NNRTI: nonnucleoside reverse-transcriptase inhibitors; NA: not applicable; *N*: total number of subject participants; 0: data status was not reported.

**Table 2:** Frequency distributions of *IL-1RN* (VNTR) and *IL-1β* (-511C/T) genotypes and alleles between HIV-infected individuals with versus without hepatotoxicity and hepatotoxicity versus healthy controls.

*IL-1RN* (VNTR) Genotypes	HIV-infected individuals with hepatotoxicity *N* = 34 (%)	HIV-infected individuals without hepatotoxicity *N* = 128 (%)	*P* value	OR (95% CI)

1/1	19 (55.9%)	60 (46.9%)	—	1 (reference)
1/2	10 (29.4%)	51 (39.9%)	0.36	0.62 (0.24–1.56)
2/2	3 (8.8%)	10 (7.8%)	0.78	0.95 (0.18–4.33)
1/3	2 (5.9%)	2 (1.6%)	0.56	3.16 (0.29–34.43)

*IL-1RN* (VNTR) Alleles	HIV-infected individuals with hepatotoxicity *N* = 68 (%)	HIV-infected individuals without hepatotoxicity *N* = 256 (%)	*P* value	OR (95% CI)

1	50 (73.52)	176 (68.75)	—	1 (reference)
2	16 (23.52)	73 (28.51)	0.50	0.77 (0.39–1.50)
3	2 (2.94)	3 (1.17)	0.68	2.35 (0.27–17.89)

*IL-1β* (-511C/T) Genotypes	HIV-infected individuals with hepatotoxicity *N* = 34 (%)	HIV-infected individuals without hepatotoxicity *N* = 128 (%)	*P* value	OR (95% CI)

CC	7 (20.6%)	21 (16.4%)	—	1 (reference)
CT	14 (41.2%)	61 (47.7%)	0.75	0.84 (0.29–2.43)
TT	13 (38.2%)	46 (35.9%)	0.47	0.68 (0.24–1.93)

*IL-1β* (-511C/T) Alleles	HIV-infected individuals with hepatotoxicity *N* = 68 (%)	HIV-infected individuals without hepatotoxicity *N* = 256 (%)	*P* value	OR (95% CI)

C	28 (41.17)	103 (40.23)	—	1 (reference)
T	40 (58.82)	153 (59.76)	0.88	1.04 (0.60–1.79)

*IL-1RN* (VNTR) Genotypes	HIV-infected individuals with hepatotoxicity *N* = 34 (%)	Healthy control *N* = 152	*P* value	OR (95% CI)

1/1	19 (55.9%)	68 (44.7%)	—	1 (reference)
1/2	10 (29.4%)	61 (40.1%)	0.30	0.58 (0.25–1.36)
2/2	3 (8.8%)	16 (10.5%)	0.78	0.67 (0.18–2.55)
1/3	2 (5.9%)	3 (2.0%)	0.69	2.39 (0.37–15.33)

*IL-1RN* (VNTR) Alleles	HIV-infected individuals with hepatotoxicity *N* = 68 (%)	Healthy controls *N* = 304 (%)	*P* Value	OR (95% CI)

1	50 (73.52%)	202 (66.44%)	—	1 (reference)
2	16 (23.52%)	95 (31.25%)	0.28	0.68 (0.37–1.26)
3	2 (2.94%)	5 (1.64%)	0.93	1.62 (0.20–8.57)

*IL-1β* (-511C/T) Genotypes	HIV-infected individuals with hepatotoxicity *N* = 34 (%)	Healthy controls *N* = 152 (%)	*P* value	OR (95% CI)

CC	7 (20.6%)	25 (16.4%)	—	1 (reference)
CT	14 (41.2%)	62 (40.8%)	0.88	0.81 (0.29–2.23)
TT	13 (38.2%)	65 (42.8%)	0.71	0.71 (0.26–2.00)

*IL-1β* (-511C/T) Alleles	HIV-infected individuals with hepatotoxicity *N* = 68 (%)	Healthy controls *N* = 304 (%)	*P* value	OR (95% CI)

C	28 (41.17%)	112 (36.84%)	—	1 (reference)
T	40 (58.82%)	192 (63.15%)	0.58	0.83 (0.49–1.42)

VNTR: variable number tandem repeat; *N*: total number of subject participants; NS: not significant; OR: odds ratio; CI: confidence interval. Odds ratio (OR) and 95% CIs were derived from logistic regression models comparing the homozygous wild-type genotype/allele (1/1 genotype and 1 allele for IL-1RN) (CC genotype and C allele for IL-1*β*) with other genotypes/alleles. Zero percent genotype frequencies in either of variables are avoided in the analysis.

**Table 3:** Frequency distribution of *IL-1RN* (VNTR) and *IL-1β* (-511C/T) genotypes/alleles in HIV-infected individuals and healthy controls.

*IL-1RN* (VNTR) Genotypes	HIV-infected individuals *N* = 128 (%)	Healthy controls *N* = 152 (%)	*P* value	OR (95% CI)

1/1	60 (46.9%)	68 (44.7%)	—	1 (reference)
1/2	51 (39.9%)	61 (40.1%)	0.93	0.95 (0.55–1.63)
2/2	10 (7.8%)	16 (10.5%)	0.56	0.71 (0.27–1.81)
1/4	3 (2.3%)	1 (0.7%)	0.54	3.40 (0.30–87.20)
1/3	2 (1.6%)	3 (2.0%)	0.87	0.76 (0.08–5.81)
2/3	1 (0.8%)	2 (1.3%)	0.90	0.57 (0.02–8.26)

*IL-1RN* (VNTR) Alleles	HIV-infected individuals *N* = 256 (%)	Healthy controls *N* = 304 (%)	*P* value	OR (95% CI)

1	176 (68.75)	202 (66.44)	—	1 (reference)
2	73 (28.51)	95 (31.25)	0.56	0.88 (0.60–1.29)
3	3 (1.17)	5 (1.64)	0.88	0.69 (0.13–3.36)
4	4 (1.56)	1 (0.32)	0.29	4.59 (0.48–108.86)

*IL-1β* (-511C/T) Genotypes	HIV-infected individual *N* = 128 (%)	Healthy controls *N* = 152 (%)	*P* value	OR (95% CI)

CC	21 (16.4%)	25 (16.4%)	—	1 (reference)
CT	61 (47.7%)	62 (40.8%)	0.62	1.18 (0.59–2.37)
TT	46 (35.9%)	65 (42.8%)	0.64	0.85 (0.43–1.68)

*IL-1β* (-511C/T) Alleles	HIV-infected individuals *N* = 256 (%)	Healthy controls *N* = 304 (%)	*P* value	OR (95% CI)

C	103 (40.23)	112 (36.84)	—	1 (reference)
T	153 (59.76)	192 (63.15)	0.46	0.87 (0.61–1.24)

*N*: total number of subject participants; OR: odds ratio; CI: confidence interval. Odds ratios (OR) and 95% CIs derived by logistic regression models comparing the homozygous wild-type genotype/allele (1/1 genotype and 1 allele for IL-1RN) (CC genotype and C allele for IL-1*β*) with other genotypes/alleles.

**Table 4:** Frequency distribution of *IL-1RN* (VNTR) and *IL-1β* (-511C/T) genotypes in different HIV disease stages of HIV-infected individuals.

Genotype *IL-1RN* (VNTR)	Healthy controls *N* = 145^∗^ (%)	Early HIV disease stage		Intermediate HIV disease stage		Advanced HIV disease stage	
		*N* = 19 (%)	OR (*P*)	*N* = 31 (%)	OR (*P*)	*N* = 71 (%)	OR (*P*)

1/1	68 (46.9)	8 (42.1)	1 (Ref.)	16 (51.6)	1 (Ref.)	36 (50.7)	1 (Ref.)
1/2	61 (42.1)	9 (47.4)	1.20 (0.74)	11 (35.5)	0.72 (0.46)	31 (43.7)	0.85 (0.65)
2/2	16 (11.0)	2 (10.5)	0.97 (0.97)	4 (12.9)	0.99 (0.99)	4 (5.6)	0.42 (0.19)

Genotype *IL-1β* (-511C/T)	Healthy controls *N* = 152 (%)	Early HIV disease stage		Intermediate HIV disease stage		Advanced HIV disease stage	
		*N* = 19 (%)	OR (*P*)	*N* = 33 (%)	OR (*P*)	*N* = 76 (%)	OR (*P*)

CC	25 (16.4)	2 (10.5)	1 (Ref.)	9 (27.3)	1 (Ref.)	10 (13.2)	1 (Ref.)
CT	62 (40.8)	11 (57.9)	2.66 (0.25)	14 (42.4)	0.64 (0.39)	36 (47.4)	1.46 (0.44)
TT	65 (42.8)	6 (31.6)	1.42 (0.69)	10 (30.3)	0.46 (0.16)	30 (39.5)	1.45 (0.44)

*N*: number of subject participants; ^∗^Individuals with rare genotypes (<5%) were excluded in the analysis; OR: odds ratio; CI: confidence interval. Odds ratios and 95% CIs were derived from logistic regression models comparing the homozygous wild-type genotype (1/1 genotype for IL-1RN) (CC genotype for IL-1*β*) with other genotypes.

**Table 5:** Frequency distribution of haplotype *IL-1RN* (VNTR) and *IL-1β* (-511C/T) in HIV-infected individuals with hepatotoxicity, without hepatotoxicity, and healthy controls.

Haplotype	HIV-infected individuals with hepatotoxicity (*N* = 66^∗^)	HIV-infected individuals without hepatotoxicity (*N* = 244^∗^)	*P* value	OR (95% CI)

1C	19 (0.28)	81 (0.34)	0.56	1 (reference)
1 T	29 (0.4)	90 (0.35)	0.25	1.41 (0.79–2.36)
2C	6 (0.09)	14 (0.05)	0.31	1.67 (0.61–4.53)
2 T	10 (0.15)	57 (0.22)	0.17	0.60 (0.29–1.25)
3C	2 (0.03)	2 (0.06)	0.15	3.85 (0.53~27.83)

Haplotype	HIV-infected individuals with hepatotoxicity (*N* = 66^∗^)	Healthy controls (*N* = 292^∗^)	*P* value	OR (95% CI)

1C	19 (0.28)	86 (0.28)	0.95	1 (reference)
1 T	29 (0.43)	111 (0.36)	0.34	1.29 (0.75–2.20)
2C	6 (0.09)	17 (0.05)	0.32	1.63 (0.61–4.31)
2 T	10 (0.15)	76 (0.25)	0.07	0.51 (0.25–1.02)
3C	2 (0.03)	5 (0.02)	0.48	1.82 (0.34~9.5)

Haplotype	HIV-infected individuals (*N* = 244^∗^)	Healthy controls (*N* = 295^∗^)	*P* value	OR (95% CI)

1C	81 (0.32)	86 (0.28)	0.44	1 (reference)
1 T	90 (0.35)	111 (0.36)	0.74	0.94 (0.66–1.33)
2C	14 (0.05)	17 (0.05)	0.95	0.98 (0.47–2.02)
2 T	57 (0.22)	76 (0.25)	0.45	0.86 (0.58–1.27)
3C	2 (0.01)	5 (0.02)	0.36	0.47 (0.09–2.45)

*N*: total number of haplotypes; OR: odds ratio; CI: confidence interval. Genotypes of *IL-RN* bearing < 5% genotype was not included in the study. Odds ratios and 95% CIs were derived from logistic regression models comparing the wild-type haplotype 1C (IL-1RN ^∗^1/IL-1*β *^∗^C) with another haplotype.

**Table 6:** Frequency distribution of *IL-1RN* (VNTR) and *IL-1β* (-511C/T) genotypes in tobacco and alcohol using HIV-infected individuals with and without hepatotoxicity.

*HIV-infected individuals with hepatotoxicity*				

*IL-1RN* (VNTR) Genotypes	Tobacco users *N* = 7 (%)	Tobacco nonusers *N* = 27 (%)	*P* value	OR (95% CI)

1/1	6 (85.7%)	13 (48.1%)	—	1 (reference)
1/2	1 (14.3%)	9 (33.3%)	0.40	0.24 (0.01–2.79)

*IL-1β* (-511C/T) Genotypes	Tobacco users *N* = 7 (%)	Tobacco nonusers *N* = 27 (%)	*P* value	OR (95% CI)

CC	2 (28.6%)	5 (18.5%)	—	1 (reference)
CT	2 (28.6%)	12 (44.4%)	0.84	0.42 (0.03–5.93)
TT	3 (42.9%)	10 (37.0%)	0.78	0.75 (0.06–9.47)

*HIV-infected individuals without hepatotoxicity*				

*IL-1RN* (VNTR) Genotypes	Tobacco users *N* = 42 (%)	Tobacco nonusers *N* = 86 (%)	*P* value	OR (95% CI)

1/1	18 (42.9%)	42 (48.8%)	—	1 (reference)
1/2	17 (40.5%)	34 (39.5%)	0.86	1.17 (0.48–2.81)
2/2	3 (7.1%)	7 (8.1%)	0.70	1.00 (0.18–5.06)
1/4	2 (4.8%)	1 (1.2%)	0.48	4.67 (0.30–139.60)

*IL-1β* (-511C/T) Genotypes	Tobacco users *N* = 42 (%)	Tobacco nonusers *N* = 86 (%)	*P* value	OR (95% CI)

CC	4 (9.5%)	17 (19.8%)	—	1 (reference)
CT	16 (38.1%)	44 (51.2%)	0.68	1.55 (0.40–6.40)
TT	22 (52.4%)	25 (29.1%)	**0.05**	**3.74 (0.97–15.57)**

*HIV-infected individuals with hepatotoxicity*				

*IL-1RN* (VNTR) Genotypes	Alcohol users *N* = 7 (%)	Alcohol nonusers *N* = 27 (%)	*P* value	OR (95% CI)

1/1	6 (85.7%)	13 (48.1%)	—	1 (reference)
1/2	1 (14.3%)	9 (33.3%)	0.40	0.24 (0.01–2.79)

*IL-1β* (-511C/T) Genotypes	Alcohol users *N* = 7 (%)	Alcohol nonusers *N* = 27 (%)	*P* value	OR (95% CI)

CC	1 (14.3%)	6 (22.2%)	—	1 (reference)
CT	3 (42.9%)	11 (40.7%)	0.84	1.64 (0.10–51.13)
TT	3 (42.9%)	10 (37.0%)	0.90	1.80 (0.11–56.75)

*HIV-infected individuals without hepatotoxicity*				

*IL-1RN* (VNTR) Genotypes	Alcohol users *N* = 43 (%)	Alcohol nonusers *N* = 85 (%)	*P* value	OR (95% CI)

1/1	21 (48.8%)	39 (45.9%)	—	1 (reference)
1/2	18 (41.9%)	33 (38.8%)	0.86	1.01 (0.43–2.38)
2/2	1 (2.3%)	9 (10.6%)	0.22	0.21 (0.01–1.81)
1/3	1 (2.3%)	1 (1.2%)	0.75	1.86 (0.0–72.41)
1/4	1 (2.3%)	1 (1.2%)	0.75	1.86 (0.0–72.41)
2/3	1 (2.3%)	2 (2.4%)	0.57	0.93 (0.0–14.35)

*IL-1β* (-511C/T) Genotypes	Alcohol users *N* = 43 (%)	Alcohol nonusers *N* = 85 (%)	*P* value	OR (95% CI)

CC	4 (9.3%)	17 (20.0%)	—	1 (reference)
CT	21 (48.8%)	39 (45.9%)	0.27	2.29 (0.61–9.28)
TT	18 (41.9%)	29 (34.1%)	0.19	2.64 (0.68–11.06)

*N*: number of subject participants; OR: odds ratio; CI: confidence interval. Odds ratios and 95% CIs were derived from logistic regression models comparing the homozygous wild-type genotype (1/1 genotype for IL-1RN) (CC genotype for IL-1*β*) with other genotypes. Significant *P* values (<0.05) and related OR and 95% CI are shown in bold.

**Table 7:** Frequency distribution of *IL-1RN* (VNTR) and *IL-1β* (-511C/T) genotypes in nevirapine and efavirenz using HIV-infected individuals with and without hepatotoxicity.

*Nevirapine usage and hepatotoxicity*				

*IL-1RN* (VNTR) Genotypes	Nevirapine users in HIV-infected patients with hepatotoxicity *N* = 23 (%)	Nevirapine users in HIV-infected patients without hepatotoxicity *N* = 116 (%)	*P* value	OR (95% CI)

1/1	11 (47.8%)	53 (45.7%)	—	1 (reference)
1/2	7 (30.4%)	48 (41.4%)	0.48	0.69 (0.23–1.94)
2/2	3 (13.0%)	10 (8.6%)	0.64	1.42 (0.33–6.20)
1/3	2 (8.7%)	1 (0.86%)	0.09	8.79 (0.71–109.16)

*IL-1β* (-511C/T) Genotypes	Nevirapine users in HIV-infected patients with hepatotoxicity *N* = 23 (%)	Nevirapine users in HIV-infected patients without hepatotoxicity *N* = 116 (%)	*P* value	OR (95% CI)

CC	4 (17.4%)	18 (15.5%)	—	1 (reference)
CT	9 (39.1%)	58 (50.0%)	0.27	0.57 (0.21–1.56)
TT	10 (43.5%)	40 (34.48%)	0.87	0.90 (0.24–3.33)

*Efavirenz usage and hepatotoxicity*				

*IL-1RN* (VNTR) Genotypes	Efavirenz users in HIV-infected patients with hepatotoxicity *N* = 11 (%)	Efavirenz users in HIV-infected patients without hepatotoxicity *N* = 12 (%)	*P* value	OR (95% CI)

1/1	8 (72.7%)	7 (58.3%)	—	1 (reference)
1/2	3 (27.3%)	3 (25.0%)	0.78	0.75 (0.10–5.56)

*IL-1β* (-511C/T) Genotypes	Efavirenz users in HIV-infected patients with hepatotoxicity *N* = 11 (%)	Efavirenz users in HIV-infected patients without hepatotoxicity *N* = 12 (%)	*P* value	OR (95% CI)

CC	3 (27.3%)	3 (25.0%)	1	Reference
CT	5 (45.5%)	2 (16.7%)	0.20	4.29 (0.47–39.40)
TT	3 (27.3%)	7 (58.3%)	0.56	1.95 (0.20–19.29)

*N*: number of subject participants; OR: odds ratio; CI: confidence interval. Odds ratios and 95% CIs were derived from logistic regression models comparing the homozygous wild-type genotype (1/1 genotype for IL-1RN) (CC genotype for IL-1*β*) with other genotypes.

**Table 8:** Frequency distribution of *IL-1RN* (VNTR) and *IL-1β* (-511C/T) genotypes in NNRTI regimen using HIV-infected individuals with and without hepatotoxicity.

*HIV-infected individuals with hepatotoxicity*				

*IL-1RN* (VNTR) Genotypes	Nevirapine users *N* = 23 (%)	Efavirenz users *N* = 11 (%)	*P* value	OR (95% CI)

1/1	11 (47.8%)	8 (72.7%)	—	1 (reference)
1/2	7 (30.4%)	3 (27.3%)	0.81	1.70 (0.26–11.84)

*IL-1β* (-511C/T) Genotypes	Nevirapine users *N* = 23 (%)	Efavirenz users *N* = 11 (%)	*P* value	OR (95% CI)

CC	4 (17.4%)	3 (27.3%)	—	1 (reference)
CT	9 (39.1%)	5 (45.5%)	0.87	1.35 (0.15–12.60)
TT	10 (43.5%)	3 (27.3%)	0.68	2.50 (0.24–28.86)

*HIV-infected individuals without hepatotoxicity*				

*IL-1RN* (VNTR) Genotypes	Nevirapine users *N* = 116 (%)	Efavirenz users *N* = 12 (%)	*P* value	OR (95% CI)

1/1	53 (45.7%)	7 (58.3%)	—	1 (reference)
1/2	48 (41.4%)	3 (25.0%)	0.46	2.11 (0.45–11.02)
1/3	1 (50.0%)	1 (50.0%)	0.60	0.13 (0.00–5.53)

*IL-1β* (-511C/T) Genotypes	Nevirapine users *N* = 116 (%)	Efavirenz users *N* = 12 (%)	*P* value	OR (95% CI)

CC	18 (15.5%)	3 (25.0%)	—	1 (reference)
CT	58 (50.0%)	2 (16.7%)	0.20	4.83 (0.59–45.70)
TT	40 (34.48%)	7 (58.3%)	0.76	0.95 (0.17–4.83)

*N*: number of subject participants; OR: odds ratio; CI: confidence interval. Odds ratios and 95% CIs were derived from logistic regression models comparing the homozygous wild-type genotype (1/1 genotype for IL-1RN) (CC genotype for IL-1*β*) with other genotypes.

**Table 9:** Frequency distribution of *IL-1RN* (VNTR) and *IL-1β* (-511C/T) genotypes in combined NNRTI regimen and alcohol using HIV-infected individuals with and without hepatotoxicity.

*HIV-infected individuals with hepatotoxicity*				

*IL-1RN* (VNTR) Genotypes	Nevirapine + alcohol users *N* = 5 (%)	Nevirapine users + alcohol nonusers *N* = 18 (%)	*P* value	OR (95% CI)

1/1	4 (80%)	7 (38.9%)	—	1 (reference)
1/2	1 (20%)	6 (33.3%)	0.63	0.29 (0.01–4.58)

*IL-1β* (-511C/T) Genotypes	Nevirapine + alcohol users *N* = 5 (%)	Nevirapine users + alcohol nonusers *N* = 18 (%)	*P* value	OR (95% CI)

CC	1 (20%)	3 (16.66%)	—	1 (reference)
CT	1 (20%)	8 (44.44%)	0.84	0.38 (0.01–20.05)
TT	3 (60%)	7 (38.88%)	0.63	1.29 (0.05–47.87)

*HIV- infected patients without hepatotoxicity*				

*IL-1RN* (VNTR) Genotypes	Nevirapine + alcohol users *N* = 37 (%)	Nevirapine users + alcohol nonusers *N* = 79 (%)	*P* value	OR (95% CI)

1/1	18 (51.43%)	35 (45.45%)	—	1 (reference)
1/2	16 (45.71%)	32 (41.56%)	0.88	0.97 (0.39–2.41)
2/2	1 (2.86%)	9 (11.69%)	0.25	0.22 (0.01–1.93)
1/4	1 (2.70%)	2 (2.53%)	0.54	0.97 (0.0–15.26)

*IL-1β* (-511C/T) Genotypes	Nevirapine + alcohol users *N* = 37 (%)	Nevirapine users + alcohol nonusers *N* = 79 (%)	*P* value	OR (95% CI)

CC	3 (8.11%)	15 (18.99%)	—	1 (reference)
CT	20 (54.05%)	39 (49.37%)	0.26	2.56 (0.59–12.68)
TT	14 (37.84%)	25 (31.65%)	0.24	2.84 (0.60–14.71)

*IL-1RN* (VNTR) Genotypes	Efavirenz + alcohol users *N* = 6 (%)	Efavirenz users + alcohol nonusers *N* = 6 (%)	*P* value	OR (95% CI)

1/1	3 (50.00%)	4 (80.00%)	—	1 (reference)
1/2	2 (33.33%)	1 (20.00%)	1.00	2.67 (0.09–129.92)

*IL-1 β* (-511C/T) Genotypes	Efavirenz + alcohol users *N* = 6 (%)	Efavirenz users + alcohol nonusers *N* = 6 (%)	*P* value	OR (95% CI)

CC	1 (16.67%)	2 (33.33%)	—	1 (reference)
CT	1 (16.67%)	1 (16.67%)	0.57	0.50 (0.00–62.81)
TT	4 (66.67%)	3 (50.00%)	0.68	4.00 (0.16–174.72)

*N*: number of subject participants; OR: odds ratio; CI: confidence interval. Odds ratios and 95% CIs were derived from logistic regression models comparing the homozygous wild-type genotype (1/1 genotype for IL-1RN) (CC genotype for IL-1*β*) with other genotypes/alleles.
